# Assessment of Pathway and Location of Posterior Superior Alveolar Artery: A Cone-Beam Computed Tomography Study

**DOI:** 10.7759/cureus.22028

**Published:** 2022-02-08

**Authors:** Riddhi Rathod, Mohit Pal Singh, Prashant Nahar, Hemant Mathur, Deeptanshu Daga

**Affiliations:** 1 Oral Medicine and Radiology, Pacific Dental College and Hospital, Udaipur, IND

**Keywords:** sinus lift procedures, radiography, pterygopalatine fossa, posterior superior alveolar artery, maxillary artery, maxillary sinus, cone-beam computed tomography

## Abstract

Objective

The current study evaluated the complicated pathway of the posterior superior alveolar artery (PSAA) and measured the prevalence, diameter, and length of PSAA to the alveolar crests of molars using cone-beam computed tomography (CBCT). The study compared findings between dentate and edentulous patients grouped by their age. In addition, the study researched the presence of the septa.

Methods

One hundred and fifty CBCT scans of patients with ages ranging from 20 to 80 years were analyzed for the study. The measurements of PSAA were obtained from CBCT scans.

Results

The PSAA was detected on CBCT scans of 87.3% of participants. The majority course of PSAA was intraosseous (right side 53.3%, left side 63.3%). The diameter of PSAA was 1.30±0.42 mm on the right side and 1.19±0.40 mm on the left side. The length of PSAA to the alveolar crest of the third molar (A1) was 17.16±2.72 mm on the right side and 17.82±3.2 mm on the left side, to the first molar (M1) was 11.6±2.66 mm on the right side and 11.65±2.37 mm on the left side, and to second molar (M2) was 12.51±1.96 mm on the right side and 12.44±2.72 mm on the left side. There was no significant difference noticed between dentate and edentulous participant groups. Six percent (6%) of the scans showed the septa in the maxillary sinus.

Conclusions

The study showed that CBCT scans and their analysis help the clinician to make a better radiographic diagnosis and clinical application while using surgical procedures, such as implant placement and sinus lift.

## Introduction

The maxillary sinus is a pneumatic space. It is the most prominent bilateral air sinus located in the body of the maxilla. It opens in the middle nasal meatus of the nasal cavity with single or multiple openings. It can be visualized on the panoramic radiograph, water’s view, computed tomography (CT), magnetic resonance imaging (MRI), and cone-beam computed tomography (CBCT) [1].

The arterial supply of the maxilla originates from the posterior superior alveolar artery (PSAA) and infraorbital artery. PSAA is the first branch of the third portion of the maxillary artery (MA) and usually arises just before the maxillary artery enters the pterygopalatine fossa. The posterior superior alveolar artery (PSAA) divides into the intraosseous branch (IObr) and extraosseous branch (EObr) before entering the posterior superior alveolar foramen. Each branch forms an anastomosis with an infraorbital artery and creates an intraosseous loop and extraosseous loop. The infraorbital artery frequently arises from a common trunk with a posterior superior alveolar artery and runs anteriorly along the maxillary sinus roof. In the maxillary posterior regions, where bone atrophy and pneumatization of maxillary sinuses are a common sequela of tooth loss, implant placement following a standard surgical protocol can significantly be challenging [2].

A very common intraoperative complication during a sinus elevation or lift procedure is the perforation of the Schneiderian membrane, which occurs in 11% to 56% of sinus elevation/lift surgeries. When a surgeon is reflecting the membrane or preparing the window to access the sinus cavity, perforation of the membrane may occur. There are specific anatomical factors involved in Schneiderian membrane perforation, including the presence of the sinus septum, lateral wall thickness, and angle between the lateral and medial walls [3]. A careful evaluation of the lateral wall thickness and PSAA is critical in pre-surgical treatment planning. This evaluation would benefit treatments such as maxillary sinus floor elevation procedure, fixation of the facial and jawbone, LeFort I surgery, orthodontic mini-screws placements, and Caldwell-Luc surgery [2].

CBCT is a reliable tool to make an anatomical distinction of the variable pattern of pneumatization impeding normal drainage of the maxillary sinus between the maxillary sinus and ethmoid-derived air-filled spaces [4]. Volumetric images of the maxilla allow visualization of the entire acquired image volume and the intimate relationship between the upper posterior teeth and the maxillary sinuses [5]. Various anatomical drainage patterns and different incidental findings provide details about the maxillary sinus and anatomy of the PSAA. And the anatomy of the PSAA includes the diameter, prevalence, course of the artery, and its relation to the maxillary sinus floor. It also helps the clinician in the future to make a better radiographic diagnosis and their clinical applications while doing a surgical procedure.

## Materials and methods

The study was prepared using 150 CBCT scans, which were taken from November 2018 to May 2020. The subjects were between the ages of 20 and 80 years, and their scans were taken for various routine investigations or treatment. The scans were acquired using the Kodak 9300 CS unit (Carestream Health Inc., Rochester, New York) machine and were processed on a computer using the Kodak dental imaging software 3D module (Version 2.4.10, Carestream Health Inc., 2007). The exposure parameters were set at 90 kVp, 12 mA, and voxel size of 300-500.

The study followed specific inclusion criteria. Good diagnostic-quality CBCT scans of patients aged between 20 to 80 years were included irrespective of gender. The CBCT scans of the patients with complete visualization of the maxillary sinus, infraorbital canal, and roof of the orbit were included. Fully erupted maxillary posterior teeth on each side of the maxilla in dentate patients' scans and the area covered from the first premolar to the second molar in partially edentulous patients' scans were included. Further, the study excluded CBCT scans showing the presence of any abnormality or pathologies in the maxillary sinus. Scans of patients showing any radiographic evidence of surgery or trauma were excluded. Scans of patients with grafted sinus, dental implants, and craniofacial abnormalities were excluded. Also, non-diagnostic CBCT scans and scans with any artifacts and faulty images were excluded.

CBCT scans with the images were loaded on 3D imaging software and were assessed to evaluate the anatomy of the maxillary sinus and course of the posterior superior alveolar artery, to determine the radiographic prevalence and diameter of the posterior superior alveolar artery, and to determine the presence of septa and health of the sinus. CBCT images were obtained with a slice thickness of 0.5 mm. The scans were grouped according to age and presence and absence of the teeth.

**Figure 1:**
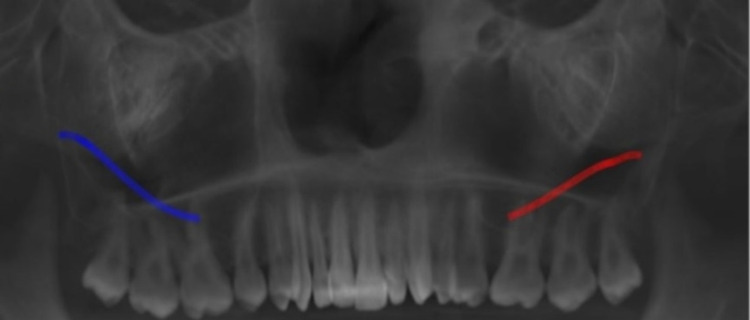
Panoramic View Showing Course of PSAA on Left and Right Side PSAA: posterior superior alveolar artery

**Figure 2:**
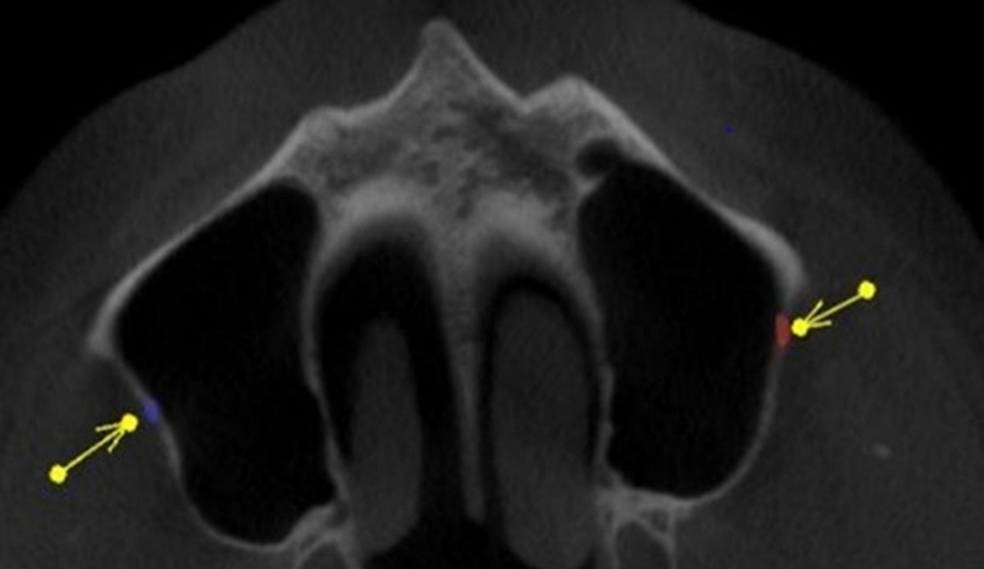
Axial View Showing the Location of PSAA on the Left and Right Sides of the Maxillary Sinus PSAA: posterior superior alveolar artery

Measurement of the lateral wall thickness was performed at three different sites, including the location of the posterior superior alveolar artery on the lateral wall of the maxillary sinus to the alveolar crest of the third molar (A1), to the alveolar crest of the first molar (M1), and to the alveolar crest of the second molar (M2). The vessel diameter was measured on the coronal section at locations corresponding to roots M1 and M2. The diameter, location, and course of the vessel from the sinus floor were evaluated to A1, M1, and M2. Figure 1 and Figure 2 shows the course and location of PSAA on the left and right side of the maxillary sinus. Superficial (on the outer cortex of the lateral sinus wall), intraosseous (inside the lateral wall), and intrasinusal (below the membrane) were the classifications used for the vessel positions.

The collected data were entered into a spreadsheet computer program. The data analysis was performed using the Statistical Package for Social Science (SPSS version 21; IBM Corp., Armonk, NY). The proportion of cases where the PSAA was identified was calculated as a total. The mean values, ranges, median values, and standard deviation of PSAA measurement were calculated and categorized by tooth position. The data were presented as mean ± standard deviation. The statistical tests applied were the paired t-test and analysis of variance (ANOVA) tests. P < 0.05 was considered statistically significant.

## Results

The study included 150 scans: 90 scans (60%) of male subjects and 60 scans (40%) of female subjects. The distribution of study participants was done according to their age and dental status. The frequency of dentate participants was 90 (60%), and the frequency of partially edentulous participants was 60 (40%). The mean age of the study participants was 41.51, and the standard deviation was 15.42. The frequency and pathway of posterior superior alveolar artery detection in the maxillary sinus were studied in 150 scans. PSAA was detected in 131 scans (87.3%) and absent in 19 scans (12.7%) as shown in Table 1.

**Table 1:** Total Number of Study Participants Based on Age, Definition, and Detection of PSAA PSAA: posterior superior alveolar artery

Age Group	Number of Patients	PSAA Detected
A1 – DENTATE 20-35 YRS	46	42
A2 – PARTIALLY EDENTULOUS 20-35 YRS	16	14
B1 – DENTATE 36-50 YRS	35	31
B2 – PARTIALLY EDENTULOUS 36-50 YRS	10	7
C1 – DENTATE 51-65 YRS	7	6
C2 – PARTIALLY EDENTULOUS 51-65 YRS	25	19
D1 – DENTATE 66-80 YRS	4	4
D2 – PARTIALLY EDENTULOUS 66-80 YRS	8	8
Total	150	131

**Figure 3:**
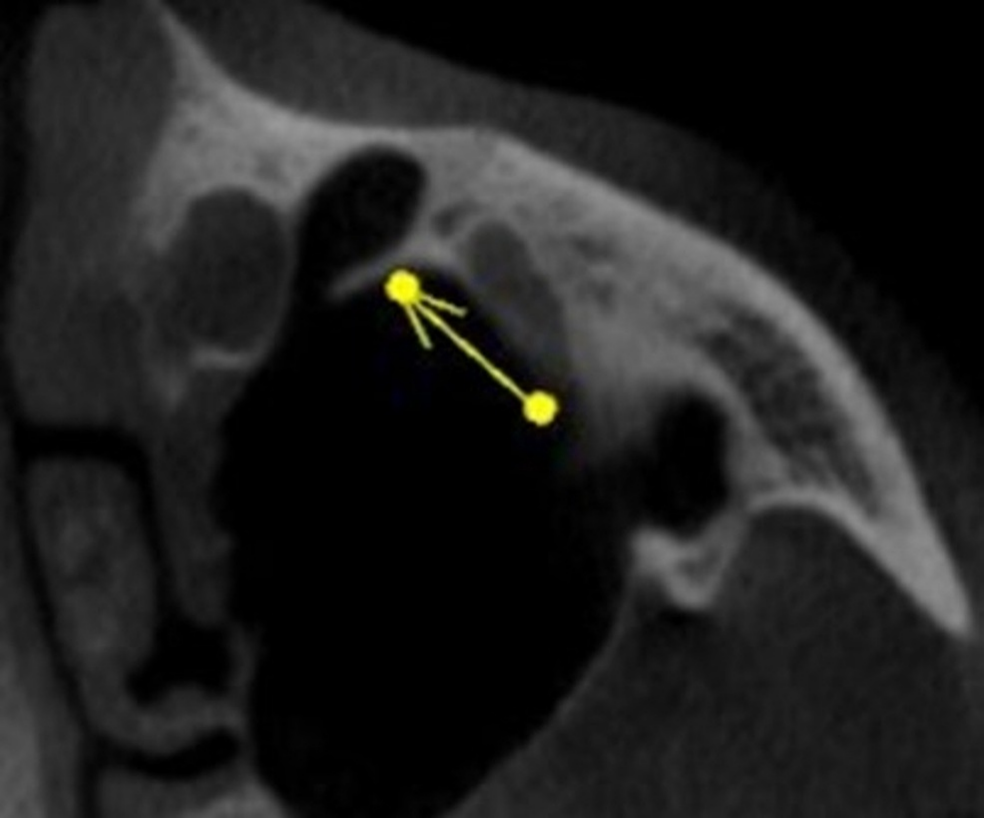
Axial View Showing the Prevalence of Septa on the Left Side

On the right side, the most common course of PSAA identified was intraosseous in 80 cases (53.3%), followed by intrasinusal, which was present in 37 cases (24.7%), followed by superficial, which was found in 15 cases (10.0%), with 132 scans (88.0%) present and undetected in 18 scans (12.0%) out of 150 total cases. On the left side, the most common course of PSAA identified was intraosseous in 95 cases (63.3%), followed by intrasinusal, which were present in 26 cases (17.3%), followed by superficial, which was found in 10 cases (6.7%). Very few scans showed the incidental finding of septa in the maxillary sinus. Figure 3 shows the prevalence of septa on the left side. The presence of septa was evident in nine scans (6.0%), and the absence of septa was evident in 140 scans (93.3%).

The evaluation of different groups showed the prevalence of PSAA based on the mean diameter value present in the maxillary sinus on the right and left sides studied in 150 scans. Table 2 shows the difference between the radiographic diameter of PSAA among different groups on the right and left sides. To measure this difference ANOVA test was applied. The mean diameter of PSAA on the right side between groups shows the p-value of 0.934, and the mean diameter of PSAA on the left side between groups showed the p-value of 1.248. These findings were found to be statistically nonsignificant. 

**Table 2:** Diameter of PSAA by Age Group PSAA: posterior superior alveolar artery

Age Group	DIAMETER OF PSAA (mm)	
	Left	Right
A1	1.13 ± 0.40	1.34 ± 0.44
A2	1.17 ± 0.51	1.11 ± 0.46
B1	1.14 ± 0.38	1.31 ± 0.36
B2	1.23 ± 0.44	1.33 ± 0.56
C1	1.27 ± 0.44	1.05 ± 0.54
C2	1.23 ± 0.34	1.34 ± 0.41
D1	1.53 ± 0.24	1.43 ± 0.15
D2	1.46 ± 0.28	1.41 ± 0.29
Average	1.19 ± 0.40	1.30 ± 0.42

The difference between length determination from different landmarks among the groups on the right and left sides, as shown in Table 3, was analyzed using the ANOVA test. The p-value of length determination from PSAA to the alveolar crest of the third molar (A1) on the right side was 0.276 and on the left side was 0.322. Both were nonsignificant. The p-value of length determination from PSAA to first molar (M1) on the right side was 0.283 and on the left side was 0.210. These values were also nonsignificant among groups. The p-value of length determination from PSAA to the second molar (M2) on the right side was 0.595 and on the left side was 0.523. All these values were found to be statistically nonsignificant among groups.

**Table 3:** Distance from PSAA to the Alveolar Crest of the Third Molar (A1), of the First Molar (M1), and of the Second Molar (M2) PSAA: posterior superior alveolar artery

Age Group	LENGTH DETERMINATION A1 (MM)		LENGTH DETERMINATION M1 (MM)		LENGTH DETERMINATION M2 (MM)	
	Left	Right	Left	Right	Left	Right
A1	17.14 ± 3.65	16.75 ± 2.70	11.95 ± 2.25	11.59 ± 2.73	12.67 ± 2.32	12.50 ± 2.28
A2	18.56 ± 2.97	18.34 ± 2.43	12.38 ± 2.56	12.98 ± 3.18	13.18 ± 2.78	13.40 ± 1.98
B1	18.34 ± 3.14	16.77 ± 2.75	11.77 ± 2.65	11.74 ± 2.76	12.12 ± 1.97	12.43 ± 1.61
B2	16.84 ± 1.76	16.49 ± 1.23	11.30 ± 1.95	11.06 ± 2.45	12.40 ± 1.61	11.85 ± 1.66
C1	17.15 ± 2.41	16.50 ± 1.61	11.27 ± 2.66	10.37 ± 1.40	12.43 ± 2.54	12.00 ± 2.21
C2	18.47 ± 2.67	17.86 ± 2.14	11.18 ± 2.31	11.33 ± 2.63	12.24 ± 2.49	12.24 ± 2.09
D1	15.78 ± 4.25	16.55 ± 3.31	12.45 ± 1.88	12.80 ± 1.86	13.53 ± 3.07	13.48 ± 1.94
D2	19.09 ± 3.14	18.58 ± 4.80	09.60 ± 1.16	10.23 ± 1.47	11.14 ± 1.57	12.51 ± 0.74
Average	17.82 ± 3.20	17.16 ± 2.72	11.65 ± 2.37	11.60 ± 2.66	12.44 ± 2.27	12.51 ± 1.96

## Discussion

**Figure 4:**
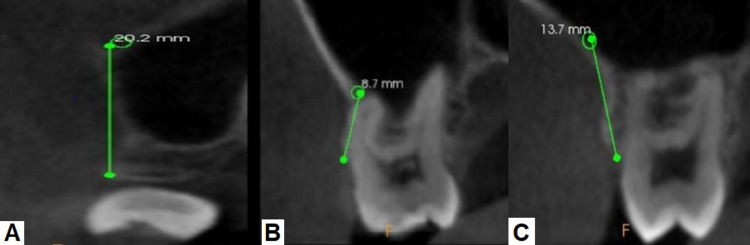
Distance From PSAA to the Alveolar Crest of the Third Molar (A1), First Molar (M1), and Second Molar (M2) A. Distance from PSAA to the Alveolar Crest of the Third Molar (A1) B. Distance from PSAA to the Alveolar Crest of the First Molar (M1) C. Distance from PSAA to the Alveolar Crest of the Third Molar (M2) PSAA: posterior superior alveolar artery

CBCT scans are being used increasingly during the past decade for diagnosis and treatment planning in dentistry. Many significant non-dental-related pathologic incidental findings have been observed via CBCT. In addition, CBCT scans very often reveal hidden results other than those related to the patient’s chief complaint. The maxillofacial radiologist or the concerned dentist must be aware of the related radiological features to prepare a correct diagnosis and consider it in the differential diagnosis [6]. The current study assessed the prevalence, course, and diameter of the posterior superior alveolar artery, presence of septa, and the health of maxillary sinus on CBCT scans. Figure 4 shows the distance of PSAA from the alveolar crest of the third molar (A1), first molar (M1), and second molar (M2).

Introduced in 1980, sinus floor elevation is a standard procedure in implant dentistry today. Maxillary sinus floor augmentation is a highly predictable method for successfully placing dental implants in the atrophic posterior maxilla. Knowledge of the anatomy of the region is essential for the success of this treatment modality [7]. The most common complication of the sinus floor elevation procedure is the perforation of the Schneiderian membrane. The complication often occurs during the membrane elevation and is associated with the presence of antral septa. Due to the presence of the maxillary sinus above the surgical site and the lack of osseous volume into which implants may project, dental implant placement in the posterior maxilla is complicated [8]. Implant placement combined with a maxillary sinus bone graft has been used widely as a predictable method with a high survival rate. It helps overcome the limitations of restoration of the posterior maxillary area with insufficient residual alveolar bone [9].

The current study included 150 CBCT scans. The scans were taken from November 2018 to March 2020 based on the inclusion and exclusion criteria as shown in Table 1. The number of scans considered for this study is comparable to the study done by Haghanifar et al., which evaluated the total number of 160 CBCT scans with the age range of 20-86 years to examine PSAA [10]. Another study by Danesh-Sani et al. evaluated the examination of PSAA and the thickness of the maxillary sinus using 430 CBCT scans. In addition, the study by Danesh-Sani et al. separated different age groups of dentate and edentulous patients, similar to the current study [3].

Further, the current study included 90 (60%) male subjects and 60 (40%) female subjects. Danesh-Sani et al. considered 239 (56%) scans of male subjects and 191 (44%) scans of female subjects [3]. In another study, Tehranchi et al. evaluated 300 patients: 138 (46%) males and 162 (54%) females [7]. The current study compared the findings between dentate patients and partially edentulous patients. Accordingly, the study included 90 (60%) of dentate patients and 60 (40%) of partially edentulous patients. The study by Danesh-Sani et al. also evaluated dentate and partially edentulous patients with the same criteria [3]. Another study by Ibrahim et al. evaluated 180 patients with the age and gender criteria and grouped them according to dentate and edentulous patients [11].

The first objective of the current study was to evaluate the prevalence, pathway of PSAA, and maxillary sinus based on age, gender, and dentition on the right and left sides [3]. The current study detected the posterior superior alveolar artery (PSAA) in 87.3% of the subjects. Danesh-Sani et al. also observed the detection rate of PSAA on CBCT at 60.58% [3]. Tehranchi et al. detected PSAA in 87% of patients on CBCT scans [7]. Ibrahim et al. observed PSAA in 73.6% of the subjects [11]. The explanation for this discrepancy among the various studies relates to several factors. Technical factors of image acquisition, such as resolution/voxel size of the radiographic system, noise, contrast, resolution, and artifacts, significantly affect image quality, which affects the identification of the PSAA. It is noteworthy to mention that the detection rate of the PSAA is 100% in cadaveric studies but highly variable in radiographic evaluation (10.5% to 93.6%) [3].

**Figure 5:**
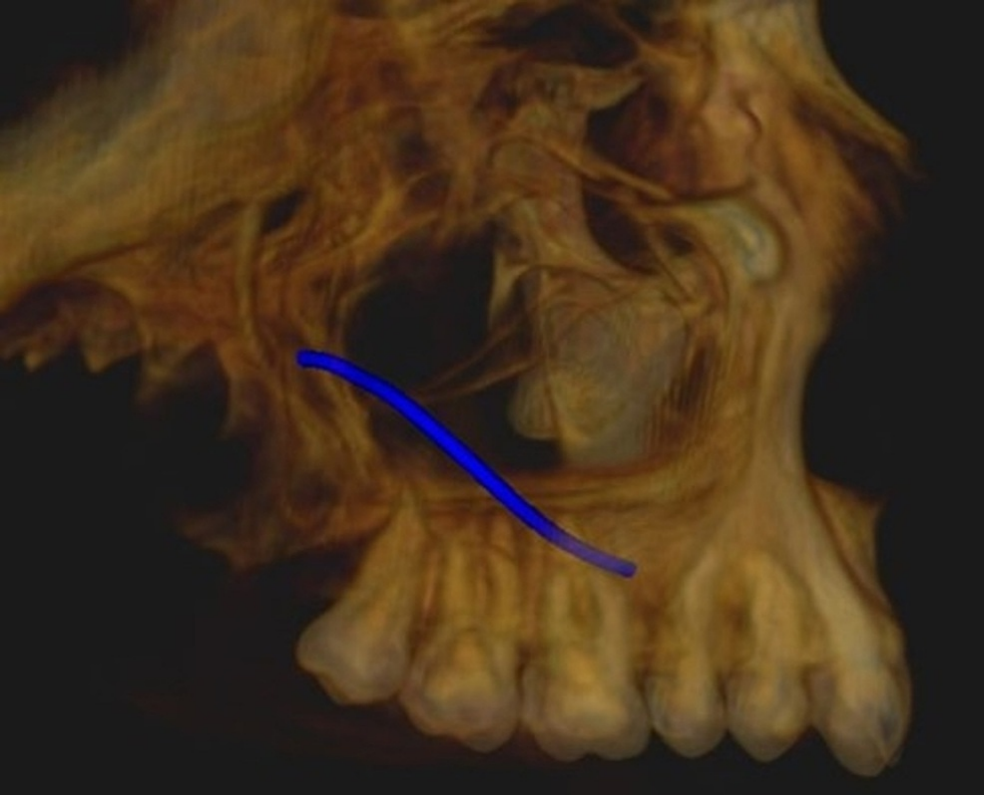
3D View Showing Pathway of PSAA on the Right Side PSAA: posterior superior alveolar artery

**Figure 6:**
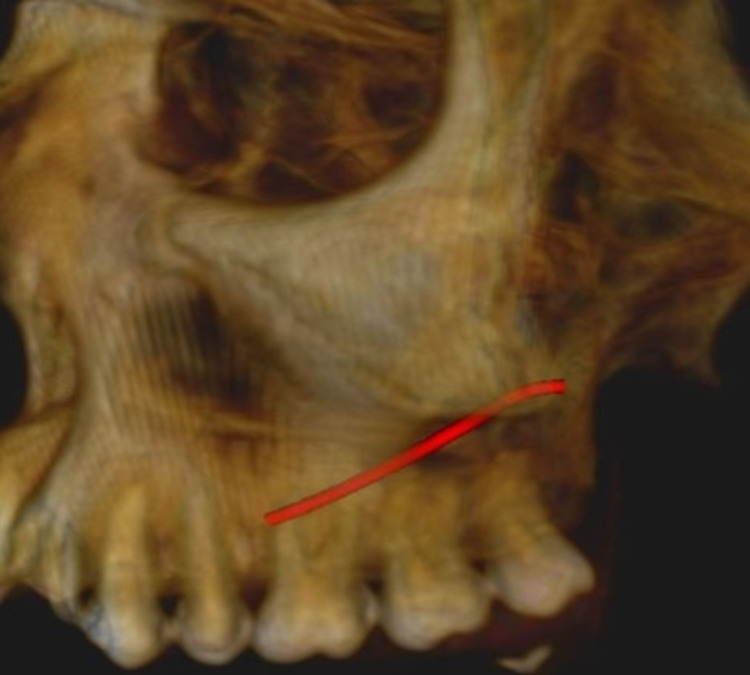
3D View Showing the Pathway of PSAA on the Left Side PSAA: posterior superior alveolar artery

On the right side, the most common course of PSAA was identified to be intraosseous in 80 cases (53.3%), intrasinusal in 37 cases (24.7%), and superficial in 15 cases (10.0%). On the left side, the most common course of PSAA was identified to be intraosseous in 95 cases (63.3%), intrasinusal in 26 cases (17.3%), and superficial in 10 cases (6.7%). Figure 5 and Figure 6 show the 3D view of the pathway of PSAA on the right and left sides, respectively. Danesh-Sani et al. observed the most frequent path of PSAA through intraosseous (69.6%), followed by intrasinusal (24.3%) and superficial (6.1%) [3]. A study conducted by Ibrahim et al. also observed that the position of the artery was mainly intraosseous in 61.94% cases below the Schneiderian membrane, and only 1.67% cases were detected on the outer cortex of the lateral sinus wall [11]. Tehranchi et al. observed the location of PSAA beneath the sinus membrane in 47% of cases and intraosseous in 47% of cases [7]. The current study’s identification of the course of PSAA on the left and right sides provides a better understanding of the course of PSAA on both sides in addition to other studies.

Out of 150 study participants, very few scans showed the incidental finding of septa in the maxillary sinus. The presence of septa was evident in nine (6.0%) scans, and the absence of septa was evident in 140 (93.3%). There was no difference detected between dentate and edentulous participants. The current study’s results showed similar frequency and presence of the septa conducted by Hadchiti et al., 348 study participants were assessed to obtain the presence of septa in the maxillary sinus. Out of 348, 37.07% showed one septum in the maxillary sinus. No difference was detected in dentate and edentulous participants [8].

This study evaluated the diameter of PSAA to be 1.30 ± 0.42 mm on the right side and 1.19 ± 0.40 mm on the left side, as shown in Table 2. The difference between the radiographic diameter of PSAA among different groups on the right and left sides was evaluated by applying the ANOVA test. The right side p-value was 0.934, and the left side p-value was 1.248. These values support that the difference between the two sides is statistically insignificant. These findings are similar to the study conducted by Danesh-Sani et al., who also observed that the mean diameter of PSAA is 1.17 mm (ranges 0.4-2.8 mm). In addition, Danesh-Sani et al. detected no significant correlation between age and the size of the PSAA [3]. Furthermore, Ibrahim et al. evaluated the diameter of the artery to be 1.15 ± 0.38mm [11]. Kim et al. reported a large diameter of 1.52 ± 0.47 mm. Guncu et al. reported the artery’s mean diameter to be 1.3 ± 0.5 mm. Moreover, Ella et al. found the mean diameter of the vessel to be 1.2 mm. These values from different studies are nearest to the current study’s results [12-14].

The current study evaluated patients between 20 and 80 years, which is similar to Güncü et al. but dissimilar to Kim et al. because Kim et al. included the patients between the ages of 45 and 65 years [12-13]. However, it is noteworthy that the results were insignificant on the ANOVA test regarding the mean diameter of the PSAA assessment in the current study. However, it was noted that the mean diameter was similar in all the groups irrespective of the variable age group, size, dental status, and comparison of the left and right sides. In comparison among all the groups by the ANOVA test, the study did not find a statistically significant relationship between the diameter of the PSAA with age, size, dental status, and left and right side.

Table 3 shows the evaluation of length determination of PSAA to the third molar’s alveolar crest (A1), first molar (M1), and second molar (M2) of different groups based on mean length values present in the maxillary sinus on the right and left sides. On the right side, the overall mean length between different groups from the location of PSAA to the alveolar crest of the third molar (A1), first molar (M1), and second molar (M2) was 17.82 mm, 11.64 mm, and 12.43 mm, respectively. On the left side, the overall mean length between different groups from the location of PSAA to the alveolar crest of the third molar (A1), first molar (M1), and second molar (M2) was 17.16 mm, 11.60 mm, and 12.50 mm, respectively. Haghanifar et al. reported the distance from PSAA to the alveolar crest of the third molar (A1), first molar (M1), and second molar (M2) to be 19.2 mm, 16.11 mm, and 16.65 mm, respectively. In addition, Haghanifar et al. evaluated the distance of the artery to the sinus floor and alveolar crest, showing no significant difference on the left and right sides [10]. In the study by Lee et al., the mean vertical height of the bony canals from the alveolar crest was 23.45 mm, 15.92 mm, and 16.61 mm at the second premolar, first molar, and second molar, respectively [2]. In another study by Park et al., the length of the PSAA to the first molar area was 16.9 mm and to the premolar area was 17.7 mm [9].

The distance of the artery decreased as it advanced towards the first molar and increased towards the premolar area. This change can be explained by (a) the anatomy of the sinus, where the sinus floor gets higher at the anterior part, and (b) the artery's course as it moves up toward the infraorbital artery [2]. In this study, the current study observed the distance of the PSAA to the alveolar crest of the first molar (M1), second molar (M2), and third molar (A1) to be similar in mean values among individuals in all the different groups with the statistical evaluation. The variability was insignificant. The mean difference between length determination from different landmarks among the group on the right and left side were statistically evaluated by the ANOVA test.

The p-value of length determination from PSAA to the alveolar crest of the third molar (A1) on the right side was 0.276 and on the left side was 0.322. Both were nonsignificant. The p-value of length determination from PSAA to first molar (M1) on the right side was 0.283 and on the left side was 0.210. These values were also nonsignificant among groups. The p-value of length determination from PSAA to the second molar (M2) on the right side was 0.595, and on the left side was 0.523. All these values were found to be statistically nonsignificant among groups. In a study by Lee et al., the mean canal height was almost similar in range with the landmarks given in the second premolar, first molar, and second molar study [2].

This study studied the health of the maxillary sinus and showed variable changes according to dentate and partially edentulous cases. In the dentate maxilla, a pyramid-shaped cavity with a base adjacent to the nasal wall and the apex pointing to the zygoma. Anteriorly, it extends to the canine and premolar area. The sinus floor usually has its most inferior point near the first molar region. The size of the sinus will increase with age if the area is edentulous. The size of the sinus increases with age if the area is edentulous [15].

Sinus lift surgery is usually performed in the edentulous maxilla when the posterior maxillary alveolus has undergone marked bone resorption. The presence of septa is pronounced in atrophic edentulous regions compared with the dentate maxilla. Maxillary sinus floor elevation via a lateral window presents complexity, often due to fragile structures and anatomical variations related to the sinus. Perforation of the Schneiderian membrane is a usual intraoperative complication during sinus elevation procedures. The presence of a thin/thick lateral wall increases the chance of membrane perforation [3].

Few limitations were noticed in this study. It would provide more in-depth details of the pathway of the PSAA and its data if a larger sample size was considered for the study. The current study observed the anatomical structure of PSAA to evaluate the pathway. Furthermore, it required a meticulous analysis of the CBCT scans to identify a pathway of PSAA. Moreover, the study was performed as a single observation study compared to a randomized control case study.

## Conclusions

The study showed no difference in the location of PSAA to the alveolar crest of the third molar (A1), first molar (M1), and second molar (M2) between dentate and partially edentulous/edentulous patients age ranging from 20 to 80 years. The study supports that it is important to evaluate the thickness of the lateral wall before surgical treatment since it may impact the integrity of the sinus membrane during the procedure. Moreover, the results suggest some factors to consider minimizing potential complications related to maxillary sinus floor augmentation.
